# Practitioner's Handbook of Diseases of the Ear and Naso-Pharynx

**Published:** 1903-03

**Authors:** 


**Practitioner's Handbook of Diseases of the Ear and Naso-pharynx.** By H. Macnaughton Jones, M.D., M.Ch.; W. R. H. Stewart, F.R.C.S. Ed.; William Milligan, M.D., C.M.; Herbert Tilley, M.D., B.S.; Ambrose Birmingham, M.D.; and Robert Dwyer Joyce, F.R.C.S.I. Sixth edition. Pp. vii., 366. London: Baillière, Tindall & Cox. 1902.

The names of the writers who have contributed to the pages of this book are alone a sufficient guarantee of its excellence. We have read no modern English book on otology better adapted for the use of practitioners and students; and it is for readers of this class, and not for specialists, that the work has been written.

The chapter on the complications of suppurative middle-ear disease, written by William Milligan, is particularly good, containing in small compass a very clear account of the symptoms, diagnosis and treatment, especially operative treatment, of these affections. We note that the author highly values the Wilde incision in the treatment of mastoid periostitis when the periostitis is primary or secondary to uncomplicated acute suppurative inflammation of the tympanic mucosa. The author states that in many such cases the inflammation within the middle ear will quickly subside after the incision has been made. Many otologists have failed to obtain these good results from a mere incision into the periosteum, and think that it is better to carry the operative procedures further, and to open the mastoid antrum.

The chapter devoted especially to diseases of the nose and naso-pharynx has been written by Herbert Tilley, and is concise and practical. We think, however, that some of the conditions discussed have rather a remote bearing on disease of the ear, and that the treatment of these by operation or otherwise will yield little or no beneficial result so far as the ears are concerned.

The volume is well got up, and is especially rich in illustrations of otological instruments.

